# Evaluation of Salivary Diagnostics: Applications, Benefits, Challenges, and Future Prospects in Dental and Systemic Disease Detection

**DOI:** 10.7759/cureus.77520

**Published:** 2025-01-16

**Authors:** Hamad Albagieh, Abdulrahman Z Alshehri, Ahmed S Alduraywishi, Albandari Aldaws, Salman S AlBalawi, Hisham F Abu Shaqqaf, Reham A Almubayi

**Affiliations:** 1 Dentistry, College of Dentistry, King Saud University, Riyadh, SAU; 2 Dentistry, Princess Nourah Bint Abdulrahman University, Riyadh, SAU

**Keywords:** biomarkers, cancer detection, dental diseases, non-invasive testing, saliva omics, salivary diagnostics, systemic diseases

## Abstract

Saliva is a multifaceted biological fluid that plays a pivotal role in oral health and overall well-being. It is primarily produced by major salivary glands, with additional contributions from minor glands. Saliva is essential for various physiological functions, including oral lubrication, digestion, and defense against pathogens. Its intricate composition comprises proteins, electrolytes, enzymes, hormones, and microbial DNA, enabling it to act as a dynamic indicator of both local and systemic health.

A literature search was conducted using PubMed, Web of Science, and Google Scholar to identify relevant studies published up to June 2024. The included studies involved human participants and provided original data or comprehensive reviews on salivary biomarkers.

The findings indicate that salivary diagnostics show promise in diagnosing and monitoring systemic conditions such as diabetes and cardiovascular diseases, with salivary glucose levels correlating well with blood glucose levels. Biomarkers such as C-reactive protein (CRP) have been linked to cardiovascular risk, while saliva has been explored for cancer detection, including pancreatic and prostate cancers. Advances in techniques such as enzyme-linked immunosorbent assay (ELISA), saliva omics, and single-cell sequencing have furthered salivary diagnostics, providing insights into disease mechanisms. Additionally, quantitative mass spectrometry (qMS) and Raman spectroscopy (RS) contribute to non-invasive diagnostics for various conditions, including cancer. Collecting saliva samples from healthy individuals is crucial for early disease detection and evaluating treatment efficacy.

This review underscores the growing importance of salivary tests in dental practice and their potential for diagnosing various health conditions. Further research is essential to address challenges related to variability and standardization. Dentists and healthcare professionals should consider incorporating salivary tests into clinical decision-making to enhance patient outcomes.

## Introduction and background

Saliva plays a crucial role in maintaining oral health and overall well-being. Primarily produced by the major salivary glands, with additional contributions from minor glands, saliva performs essential physiological functions, including lubrication, digestion, and protection against microbial infections. This multifaceted fluid contains various molecular and microbial substances that can serve as indicators of both local and systemic health conditions, making salivary diagnostics a valuable tool in healthcare [1].

Salivary dysfunction, characterized by decreased saliva production (hyposalivation), is commonly observed in clinical settings and can significantly impact patients' quality of life. Symptoms such as discomfort, pain, difficulty speaking or swallowing, and an increased risk of dental caries often accompany inadequate saliva flow. Additionally, hyposalivation alters the concentration and composition of salivary biomarkers. For example, reduced salivary flow is often associated with lower levels of antimicrobial peptides and higher concentrations of inflammatory markers, which can influence the diagnostic accuracy of saliva-based tests [2,3].

The advantages of saliva as a diagnostic medium lie in its non-invasive collection process, which is simple, painless, and cost-effective compared to blood sampling. These attributes contribute to increased patient compliance and facilitate broader clinical applications. Recent advancements in salivary diagnostics have expanded its potential beyond traditional dentistry, enabling the early detection and management of systemic conditions such as cardiovascular diseases and diabetes, thereby improving patient outcomes [4].

Despite its promise, the integration of salivary diagnostics into routine clinical practice faces challenges. Variations in saliva composition due to factors such as age, gender, diet, and medication can impact the precision and reliability of diagnostic results. Furthermore, the absence of standardized protocols for sample collection, preparation, and analysis hampers widespread clinical adoption. Overcoming these challenges through robust research and the development of standardized methods is essential for advancing this technology [4].

This review aims to provide a comprehensive overview of the role of salivary diagnostics in modern medical and dental practice. It highlights the potential of salivary biomarkers in enhancing disease detection and monitoring through non-invasive, cost-effective testing methods. By exploring the dynamic relationship between salivary constituents and systemic health conditions, this study emphasizes the importance of saliva as a valuable tool in the evolving landscape of diagnostic medicine.

## Review

Methodology

A comprehensive literature search was performed using PubMed, Web of Science, and Google Scholar to identify relevant studies published up to June 2024. The PICO question guiding this review is: In patients undergoing diagnostic evaluation (Population), how effective are salivary diagnostic methods (Intervention) compared to traditional diagnostic approaches (Comparison) in improving early detection, diagnostic accuracy, and patient outcomes (Outcome)? The search terms included "saliva", "salivary diagnostics", "oral fluids", "dental practice", and "biomarkers". The search was limited to articles written in English that involved human subjects. To expand our database, manual searches of the references cited in the selected studies were also conducted to uncover additional pertinent literature. The inclusion criteria were designed to select studies that focused on the diagnostic applications of salivary biomarkers in dental and medical practice. Only studies involving human participants and presenting original research data or comprehensive reviews were considered. Exclusion criteria were set to omit studies not directly related to the clinical application of salivary diagnostics, studies involving animal models, and those lacking rigorous methodology.

Salivary biomarkers

The studies identified a variety of salivary biomarkers, including enzymes, proteins, hormones, antibodies, and genetic material. Numerous studies have investigated the function of enzymes such as matrix metalloproteinases (MMPs) in periodontal disease, demonstrating their capacity to distinguish between healthy and diseased periodontal tissues. Studies such as Ramseier et al. (2016) and Kulhavá et al. (2020) [5,6] assessed the relationship between dental caries and proteins such as histatin and statherin, suggesting these proteins could potentially serve as predictive markers for caries risk.

The study by Ramseier et al. was a clinical observational study aimed at evaluating the ability of host- and microbially derived biomarkers to identify periodontal disease status using whole saliva and plaque biofilm. The study included 100 human subjects divided into healthy/gingivitis and periodontitis groups. Salivary biomarkers such as MMP-8 and MMP-9, along with bone turnover markers, were assessed using antibody arrays, while the presence of periodontal pathogens such as *Porphyromonas gingivalis* (*P. gingivalis*) and *Treponema denticola* was quantified using quantitative polymerase chain reaction (qPCR). The results showed elevated levels of MMP-8 and MMP-9 in advanced periodontitis patients, with the combination of salivary biomarkers and biofilm data improving disease prediction (area under the curve (AUC) = 0.88; odds ratio = 24.6). However, limitations included its cross-sectional design, which restricted the ability to assess causality or predict longitudinal changes, as well as potential variability in host responses and oral hygiene habits affecting the findings. The sample size, although adequate, may limit generalizability to diverse populations [5].

Similarly, the study by Kulhavá et al. was an experimental cross-sectional study that analyzed differences in protein abundance in saliva between 27 male participants (15 caries-positive and 12 caries-free). The study identified calcium-binding proteins and immune proteins in relation to caries resistance, with 14 proteins in the supernatant being significantly upregulated in the caries-free group, indicating their role in caries prevention, while three proteins in the pellet were more abundant in the caries-positive group, suggesting their role in enamel decalcification. Limitations of this study included its small sample size, the inclusion of only male participants, and its cross-sectional design, making it difficult to determine whether protein differences preceded or followed the onset of caries [6].

Both studies underscore the potential of salivary diagnostics in identifying biomarkers for oral diseases, with Ramseier et al.'s research highlighting the importance of combining host and microbial biomarkers for periodontal disease prediction and Kulhavá et al.'s study shedding light on specific protein compositions related to caries resistance [6]. The limitations of both studies emphasize the need for larger, longitudinal research to validate these findings and improve clinical applicability. Researchers also looked at biomarkers such as interleukins and cytokeratins in the context of oral cancer and found that they could help with the early detection and monitoring of the disease [7].

Certain biomarkers discovered in saliva have demonstrated great sensitivity and specificity, especially in oral disorders such as periodontal disease, dental caries, and oral cancer. However, detecting disease-specific molecular biomarkers in total saliva is challenging due to the advanced technologies required. The type of saliva utilized for diagnostic purposes also affects detection. For instance, unstimulated saliva includes larger amounts of screening/diagnostic biomarkers compared to stimulated saliva [5,6].

Most of the studies on the association between salivary biomarkers and oral and systemic disorders have various methodological limitations, as many are cross-sectional with a small sample size. Consequently, establishing causal associations between studied biomarkers in saliva and specific conditions is difficult. The existing knowledge regarding salivary biomarkers and disease diagnostics remains inadequate, thus necessitating the development of non-invasive screening and diagnostic approaches [6,8].

Xerostomia and Sjögren's syndrome

Xerostomia caused by polypharmacy requires prescription adjustments to products with fewer xerogenic side effects, as well as reducing the number of prescribed medications, which can be challenging for physicians and pharmacists [9]. Topical medicines used to treat dry mouth caused by chronic inflammatory disorders, (auto-)immunological or granulomatous diseases, and radiation therapy often fail to account for overnight xerostomia and natural salivary characteristics, making them frequently unsuccessful. Although few pharmacological agents are available, intravenous injections have side effects, and oral administration with intestinal absorption requires swallowing tablets, which can be difficult for patients and associated with side effects. There is an urgent need for research in this field to improve the treatment and current standard of care [9].

Sjögren's syndrome presents in various ways, starting with local involvement of exocrine glands with keratoconjunctivitis sicca and reduced saliva production and extending to systemic involvement affecting multiple organs. This systemic impact significantly impairs patients' quality of life. The diagnosis of Sjögren's syndrome involves three goals: identification of evidence of dry eyes, dry mouth, and confirmation of autoimmunity. In diagnosing Sjögren's syndrome, a gum test can evaluate oral dryness (salivary flow), with a cutoff value of 1.0 mL/minute for stimulated salivary flow and a recommended value of 1.4 mL/minute when using flavored gum [10,11].

Local lymphocytic infiltration of the exocrine gland is the histological feature of this syndrome. Laboratory tests used for diagnosis include markers such as anti-nuclear antibodies, anti-Sjögren's syndrome type A (anti-SS-A), anti-SS-B, cryoglobulins, and hypocomplementemia. These antibodies may cross the placenta during pregnancy, causing issues for the fetus, such as myocarditis, arrhythmia, and impaired clearance of apoptotic cells. The impact of Sjögren's syndrome on salivary glands can be assessed with recent modalities such as sialography, MRI, and salivary gland ultrasonography [10,11].

Periodontal disease

Periodontal diseases are among the most prevalent oral diseases worldwide, caused by interactions between the host and oral bacteria. Metabolites, which are intermediate products of metabolism, play important roles in understanding the pathogenesis of periodontal diseases and can help in the early detection and prognosis of oral diseases [12].

Several studies have explored the use of salivary diagnostics for periodontal disease, focusing on biomarkers such as inflammatory cytokines, enzymes, and microbial components. For example, a study demonstrated that biomarkers such as MMP-8 and interleukin-1β (IL-1β) are significantly elevated in saliva in patients with periodontal disease compared to healthy individuals. The risk of severe periodontal disease is higher, with a salivary flow rate below 1.4 mL/minute, while a flow rate of 2.7 mL/minute is considered a risk threshold for teeth affected by periodontal disease [5].

*P. gingivalis* represents a keystone pathogen that disrupts immune responses but is not a direct inducer of inflammation. Diagnostic kits such as the My Perio Path kit can help identify bacteria and assess associated risks, aiding in diagnosis and treatment planning for associated diseases such as diabetes, heart disease, and pregnancy complications [13].

Dental caries

Many factors contribute to dental caries, leading to the demineralization of the hard tissues of the teeth. Cariogenic bacteria produce acids that contribute to caries formation. Saliva can help protect and maintain oral health through antibacterial effects. To better evaluate susceptibility to dental caries, awareness about the importance of salivary tests should be increased [14].

Most studies analyzed protein biomarkers in the diagnosis of dental caries, while some also assessed metabolites or gene biomarkers. Several potential salivary biomarkers for dental caries have been identified, including proteins (e.g., mucins, alpha-amylase, interleukins), metabolites (e.g., urea), and genes (e.g., carbonic anhydrase). Saliva shows promise as a non-invasive biofluid for early diagnosis, with many identified biomarkers being useful for diagnosis or predicting risk [15].

Various salivary biomarkers, including antimicrobial peptides, enzymes, and glycoproteins, are associated with dental caries. Increased levels of antimicrobial peptides such as LL-37, statherin, and histatins are linked to lower caries risk, while increased levels of enzymes such as alpha-amylase and carbonic anhydrase are linked to a higher risk. However, the association between glycoproteins (e.g., mucins, immunoglobulins, proline-rich proteins) and caries risk has been mixed [16].

CD14 is a principal molecule in the activation of innate immune cells, existing in both membrane-anchored and soluble forms. Higher CD14 concentrations are found in children without caries compared to those with caries, and increased CD14 levels help prevent caries by regulating immune responses. In addition, the level of salivary IgA is lower in people with high dental caries compared to those with lower caries risk [17,18].

Systemic diseases

Saliva has been investigated for its potential to diagnose and monitor systemic diseases. For instance, salivary glucose levels have been evaluated as a non-invasive alternative to blood glucose monitoring in diabetic patients. A significant correlation between salivary and blood glucose levels was found, suggesting that saliva could serve as a viable medium for diabetes management [19]. A systematic review examining the relationship between salivary biomarkers and cardiovascular diseases (CVDs) found that salivary C-reactive protein (CRP) shows consistent increases in CVD patients [20].

Cardiovascular diseases

CVDs are another area where salivary diagnostics are gaining traction. Saliva contains biomarkers reflective of systemic inflammation and endothelial dysfunction, which are hallmarks of CVDs. Elevated salivary CRP levels could help identify individuals at high risk of myocardial infarction and stroke, providing a non-invasive method for early risk assessment [21]. CVDs, including atherosclerosis, coronary heart disease, and myocardial infarction, are the leading causes of death worldwide. Risk factors include tobacco use, high blood pressure, high blood glucose, lipid abnormalities, obesity, and physical inactivity. Salivary markers of CVDs include CRP, myoglobin (MYO), creatinine kinase myocardial band (CK-MB), cardiac troponins (cTn), and myeloperoxidase. Salivary CRP levels were found to correlate with plasma CRP levels, and salivary MYO levels were positively correlated with their serum concentrations. Although CK-MB and troponins are detected in saliva, they have limited diagnostic value [22,23]. The simplicity and non-invasive nature of saliva collection make it advantageous over other biological fluids [20].

Diabetes mellitus

The relationship between salivary composition and diabetes mellitus has been extensively studied. Salivary glucose concentrations correlate well with blood glucose levels, making saliva a potential medium for diabetes monitoring. Studies have demonstrated that salivary glucose, along with biomarkers such as amylase and cortisol, can provide insights into glycemic control and diabetic complications [24]. These biomarkers help monitor blood sugar levels and offer clues regarding the patient's stress and metabolic state, which are crucial for diabetes management. There is a significant relationship between salivary glucose concentration and glycemia/HbA1c values, with the correlation increasing at higher glycemia/HbA1c levels [25]. Additionally, chromogranin A, found in secretion granules of nerve, endocrine, and immune cells, has high gene expression in diabetic patients. Potassium, total protein, and secretory IgA levels were higher in diabetic patients than in healthy individuals, while the mean sodium and amylase values were higher in the healthy group [26,27].

Infectious diseases

Saliva has played a crucial role in the diagnosis and monitoring of several infectious diseases. During the COVID-19 pandemic, saliva-based testing emerged as a dependable and less invasive alternative for nasopharyngeal swabs. Studies have indicated that saliva testing for SARS-CoV-2 demonstrates comparable sensitivity and specificity to traditional testing methods. This makes saliva an attractive option for widespread testing due to its non-invasive collection method, enhancing patient compliance, especially in populations where traditional methods pose challenges [28,29]. Saliva has also been used to detect other infectious agents, such as HIV, hepatitis viruses, and bacterial pathogens associated with respiratory infections. Salivary interferon-gamma levels have been correlated with oral complications in HIV-infected individuals and may also help in diagnosing tuberculosis [30,31].

Cancer detection

Cancer is a worldwide health problem, and many types of cancer show no symptoms in their early stages. Salivary tests have assisted healthcare providers in the early detection of cancer. For example, a combination of four messenger RNA biomarkers, Kirsten rat sarcoma viral oncogene homolog, methyl-CpG binding domain protein 3-like 2, acrosomal vesicle protein 1, and dolichyl-phosphate mannosyltransferase subunit 1 (KRAS, MBD3L2, ACRV1, and DPM1), found in saliva can differentiate pancreatic cancer patients from non-cancer subjects [32,33]. Physicians can use salivary biomarkers for detecting other cancers, such as prostate-specific antigens for prostate cancer, human epidermal growth factor receptor 2 (HER2/neu) for breast cancer, and cancer antigens for other malignancies. Saliva is promising for diagnosing oral squamous cell carcinoma (OSCC), and further research is needed to confirm the sensitivity and specificity of identified biomarkers [34]. Biosensors for detecting various salivary compounds are becoming increasingly important in general pathology, dentistry, and pharmacology [35]. Smokers have increased levels of certain salivary biomarkers associated with periodontal disease, while other biomarkers are found to be decreased, showing the varying impact of smoking on oral health [23].

Certain salivary miRNAs, such as upregulated miRNA 184 and downregulated miRNAs 21 and 145, can serve as potential biomarkers to predict malignancy in oral potentially malignant disorders (OPMD) [36]. Studies suggest further analysis in different populations to validate these diagnostic and prognostic potentials. Additionally, pro-inflammatory cytokines in saliva, including IL-1β, IL-2, IL-6, and tumor necrosis factor-alpha (TNF-alpha), are associated with the severity of oral mucosal tissue damage in cancer patients and may serve as early markers for graft-versus-host disease [30,37].

Salivary diagnostic test

Salivary fluid, as a biological fluid, offers numerous diagnostic possibilities. Due to its effects on oral health, the use of salivary fluid as a diagnostic tool is likely to increase, involving dentists more in diagnosing and monitoring non-oral illnesses [14]. Understanding the diagnostic tools to identify oral diseases is crucial to prevent complications such as poor quality of life and low self-esteem. Salivary measurements are an important tool for identifying oral diseases. Major salivary glands secrete most of the saliva, which plays a key role in oral health, while minor salivary glands contribute to saliva production to a lesser extent.

Saliva has multiple benefits for oral health, including its lubricating properties, formation of a protective pellicle around the enamel, and role in accelerating the healing of injured soft tissue. It also aids in chewing and swallowing food [35]. Collecting saliva is simple, cost-effective, and does not require advanced training compared to serum collection. People can collect samples at home, and saliva requires less manipulation during diagnostic procedures [9]. However, saliva has some drawbacks, such as lower biomarker concentrations (10-1500 times lower than in plasma) and changes in flow rate under stress, exercise, and during digestion [12].

Saliva can be collected through two methods: unstimulated flow (e.g., passive drool) and stimulated flow (e.g., chewing gum, swabs, lozenges). Each method presents challenges, such as pH changes or contamination, which may impact biomarker stability [38].

New technology in saliva detection

Traditional saliva detection techniques, such as amylase test strips, two-dimensional electrophoresis, and mass spectrometry, have limitations such as poor specificity and false positives/negatives [39,40]. Among these, liquid chromatography (LC) combined with mass spectrometry (LC-MS) requires intensive laboratory work and a large sample content, often leading to poor repeatability [41]. Today, more advanced saliva detection methods include Raman spectroscopy (RS), enzyme-linked immunosorbent assay (ELISA), surface-enhanced laser desorption/ionization time-of-flight mass spectrometry (SELDI-TOF-MS), quantitative mass spectrometry (qMS), saliva kits, omics, and single-cell sequencing technology [40].

Saliva creatinine

Salivary glands allow urea nitrogen to diffuse from blood into saliva, making saliva a potential diagnostic indication for kidney disease. Salivary urea and creatinine levels are higher in renal disease patients than in healthy individuals, correlating with disease severity [42,43]. End-stage renal disease (ESRD) treatments such as dialysis can alter saliva's functions, affecting lubrication, antibacterial activity, and digestion [9]. Studies have shown notable variations in urea, creatinine, potassium, and chloride levels in patients' saliva [44].

Heavy metals in saliva

Heavy metals (e.g., zinc, copper, iron, cobalt, arsenic, cadmium, mercury) are used in dental treatments such as orthodontic appliances and crowns. These metals biodegrade in the oral environment, which could have cytotoxic effects [9,43]. Orthodontic treatments have shown that nickel, chromium, and silver inhibit fibroblast proliferation. Saliva accumulation of heavy metals can cause inflammation, oxidative stress, cytokine production, and DNA damage. Various metal ions, such as titanium, chromium, cobalt, and nickel, are also present in dental restorations. Elevated levels of metal ions have been linked to mutations, cell death, chronic inflammation, and hypersensitivity reactions [13,45-47]. Additionally, studies have found that in human primary monocytes, Ni^2+^ stimulates the production of inflammatory genes, including interleukin-8 (IL-8) [48].

SDS-PAGE (sodium dodecyl sulfate-polyacrylamide gel electrophoresis) of saliva

SDS-PAGE is a widely used laboratory technique that separates proteins based on their molecular weight. In the context of saliva, SDS-PAGE is used to analyze the protein composition by denaturing the proteins and applying an electric current to separate them through a polyacrylamide gel matrix. This method helps in identifying and characterizing salivary proteins, including enzymes, antibodies, and other bioactive molecules, which can be useful in diagnosing oral and systemic diseases, studying salivary biomarkers, and understanding the proteomic profile of saliva [49].

Two-dimensional electrophoresis (2D PAGE), introduced in 1975, is another biochemical technique for dividing complex protein mixtures into separate species. SDS-PAGE is used after isoelectric focusing to separate proteins by size. Autoradiography, protein staining, or Western blotting can be used to observe the resulting protein spots [45]. For example, multiple isoforms of Zn-α-2 glycoprotein have been identified in saliva, potentially linked to lipid metabolism, high serum levels, and prostate cancer [46]. The synthesis of parotid protein fragments may be affected by oral diseases, making them potential biomarkers. Salivary protein and peptide coding analysis has also identified hormones, cytokines, growth factors, and peptides with significant biological activities in plasma and cerebrospinal fluid [47].

ELISA (enzyme-linked immunosorbent assay)

ELISA is a laboratory technique used to detect and quantify specific proteins, antigens, antibodies, or other molecules in a sample. This highly sensitive and specific method is commonly employed in research, diagnostics, and various clinical applications. ELISA is widely used for quantifying chemicals in biological fluids as well as antigen-antibody responses through color changes produced with enzyme-linked conjugates and substrates [50].

Brain-derived neurotrophic factor (BDNF) was measured by ELISA in both saliva and plasma. During acute stress, BDNF in the submandibular gland has been shown to influence plasma BDNF levels. In patients with type 2 diabetes, ELISA is used to assess oxidative stress in saliva. Moreover, studies have demonstrated correlations between stress and insulin resistance markers (e.g., cortisol, insulin, adiponectin, resistin) in blood and saliva [51,52].

Saliva omics

Saliva omics focuses on the identification of all "omics" components, including DNA, RNA, proteins, metabolites, and microbiota. It encompasses different areas such as genomics, transcriptomics, proteomics, epigenomics, metabolomics, and microbiomics and has emerged as a novel approach for saliva detection [39].

This concept allows a comprehensive study of salivary components, contributing to a better understanding of oral and systemic health, identifying potential biomarkers, and providing valuable insights into diagnostics.

Proteomics

Salivary detection-based research has grown rapidly with advancements in genomic technologies and omics research. Proteome analysis is mainly utilized to differentiate between normal and pathological states of disease. Initial research has been conducted on several illnesses, including Sjögren's syndrome, chronic graft-versus-host disease, OSCC, and oral leukoplakia [53].

Additionally, the salivary proteome has been used to assess immune-mediated inflammatory systemic diseases, including multiple sclerosis [54], diabetes, cystic fibrosis, and Parkinson's disease [23,55-57]. This evaluation demonstrates the potential of proteomics in identifying biomarkers and offering insights into the molecular mechanisms underlying certain systemic diseases.

Metabolomics

Metabolomics is an approach that characterizes the illness phenotype by analyzing small molecules (metabolites) within cells, tissues, or organisms. It connects all "omics" approaches by providing insight into cellular processes and physiological changes. In metabolomics, nuclear magnetic resonance (NMR) and mass spectrometry are the most commonly used methods [58].

NMR metabolomics has clarified the metabolic pathways linked to various local and systemic illnesses, such as tooth caries, fatty arthritis, type 1 diabetes, melanoma, and oral cancer [59,60]. It has also been widely used to better understand the COVID-19 phenotype and how SARS-CoV-2 affects physiological aspects. NMR metabolomics is particularly promising for identifying biomarkers with diagnostic potential and studying metabolic pathways related to smell and taste disorders in COVID-19 patients [58].

Single-cell sequencing

Single-cell sequencing has advanced rapidly in recent years, offering high-resolution insights into structural, copy number, and single nucleotide variations of individual cells [61]. It has proven valuable in preparing human cell profiles for cancer research, enabling efficient management and personalized treatment. Single-cell sequencing helps locate and analyze the immune microenvironment, tumor heterogeneity, circulating tumor cells, rare subpopulations, and molecular subtypes in cancer research. These are closely related to processes such as tumorigenesis, progression, metastasis, recurrence, drug resistance, and cancer stem cells [62,63].

Single-cell sequencing has also been used in studies on African trypanosomes (*Trypanosoma brucei*), which are transmitted through the bite of infected tsetse flies. Diagnosing and treating these infections in isolated areas remains challenging [64]. Researchers isolated *Trypanosoma brucei* from 2,045 parasites obtained from infected tsetse fly salivary glands using single-cell RNA sequencing (scRNA-seq). They discovered that each parasite expressed a distinct variant surface glycoprotein (mVSG), which may serve as a target for vaccine development to block transmission [64].

Single-cell sequencing has clinical significance because it provides insight into dynamic cellular metabolism and transcription, advancing our understanding of disease mechanisms and enabling the development of targeted therapies.

Saliva kits

Saliva kits are emerging as reliable tools for detecting various diseases, including AIDS. In China, a new salivary AIDS detection product has demonstrated accuracy rates of over 99%, making it suitable for rapid, accurate, and painless testing [65,66]. These kits work as in vitro immunodiagnostic reagents, allowing qualitative detection of HIV1/2 antibodies in oral secretions through visual interpretation of results. However, the test results should be used in conjunction with other diagnostic tools, as they cannot be used as the sole basis for a definitive diagnosis [31].

Saliva kits represent a significant advancement in disease diagnostics by offering non-invasive and accessible solutions for various health conditions. They have the potential to be used for self-testing and screening in resource-limited settings, improving accessibility to healthcare.

Quantitative mass spectrometry (qMS)

qMS is an advanced analytical technique used to measure the quantity of specific molecules, such as proteins or metabolites, in a complex biological sample. Unlike traditional mass spectrometry, which primarily identifies the presence of molecules, qMS provides a quantitative aspect, allowing researchers to determine the absolute or relative abundance of these molecules [67].

In this technique, the measured material is ionized and separated based on its ionic mass-to-charge ratio, and the peak intensities of different ions are detected. qMS is often used in proteomics to measure differences in the physiological states of biological systems. One significant application of qMS is measuring cortisol levels in biological samples, including saliva, urine, and serum, as cortisol serves as an endocrine stress indicator [68].

For quantification, qMS often uses differential stable isotope labeling to create a mass marker recognizable by the mass spectrometer. Through metabolism, chemistry, enzymes, or the inclusion of synthetic peptide standards, these mass markers are added to proteins or peptides [67].

Quantitative analysis can also be achieved through label-free quantification techniques, correlating the peptide sequence numbers, relative or absolute protein quantities, and proteolytic peptide mass spectrometry signals directly [67]. This quantification capability is a key advancement in proteomics, as it provides insights into molecular-level changes in various disease states, enabling further understanding and identification of potential biomarkers [67].

Raman spectroscopy (RS)

RS is a technique that relies on the interaction between photons and vibrating atoms in a molecule, followed by inelastic scattering. Photons are excited to a virtual energy state using monochromatic light, typically in the visible, ultraviolet (UV), or near-infrared (NIR) range. The ensuing energy loss (Stokes) or gain (anti-Stokes) is then measured through inelastic scattering, also known as the Raman effect. This energy transfer reveals a discrete vibrational pattern of polar molecules, allowing for qualitative assessments of biological compositions [69,70].

However, biological samples often exhibit weak Raman scattering, which can overlap with background signals from cellular components. To improve the Raman signal, surface-enhanced Raman scattering (SERS) was developed, which overcomes these limitations and allows for better detection [70]. RS is highly specific at the molecular level and can detect disease-induced molecular alterations, which enables extremely precise biochemical fingerprinting of cells and tissues for cancer diagnosis [70,71].

In saliva, RS and SERS can be used to detect various biomolecules, such as proteins, nucleic acids, and enzymes, which vary between cancer patients and healthy individuals. These biomarkers have shown potential for cancer detection, as the SERS spectral signature of saliva can reflect changes in proteins and metabolites occurring during treatment and disease progression [72]. RS and SERS have been investigated as non-invasive diagnostic tools for detecting diseases such as nasopharyngeal carcinoma [73], OSCC [51], lung cancer [74], Sjögren's syndrome [75], and benign versus malignant breast tumors [76,77].

Surface-enhanced laser desorption/ionization time-of-flight mass spectrometry (SELDI-TOF-MS)

SELDI-TOF-MS is an effective technique for detecting low-molecular-weight proteins (less than 15 kilodaltons, kDa) with high sensitivity. This method is particularly useful for identifying biomarkers that might be missed by other proteomic techniques, such as two-dimensional gels [68,78]. One major advantage of SELDI-TOF-MS is that it does not require extensive sample purification or protein separation before analysis. Because biomarker proteins generally account for less than 1% of the total plasma protein, mass spectrometry is a more sensitive technique for detecting these markers compared to other methods [79,80].

The sensitivity of SELDI-TOF-MS makes it ideal for cancer detection, as it allows the use of low-protein tissue extracts or saliva as sample materials. Furthermore, SELDI-TOF-MS has demonstrated reliability and sensitivity during repeated testing [81]. When paired with radiological data, SELDI-TOF-MS can identify internal cancers before symptoms manifest by detecting specific protein alterations that might indicate the presence of cancerous tissues. This makes it a powerful tool for early diagnosis and intervention.

Table 1 presents the summary of key studies on salivary biomarkers and diagnostic applications in oral and systemic diseases.

**Table 1:** Summary of key studies on salivary biomarkers and diagnostic applications in oral and systemic diseases

Study Topic	Key Findings	References
Salivary Biomarkers in Periodontal Disease	Matrix metalloproteinases (MMPs) can distinguish between healthy and diseased tissues.	[5,6]
Biomarkers for Dental Caries	Proteins like histatin and statherin show predictive potential for caries risk.	[5,6,14,15]
Oral Cancer Detection	Salivary interleukins and cytokeratins aid in early detection of oral squamous cell carcinoma (OSCC).	[7,34]
Xerostomia and Sjögren’s Syndrome	Sjögren’s syndrome affects exocrine glands, leading to reduced saliva flow and systemic complications.	[9,10,11]
Periodontal Disease and Bacterial Pathogens	Elevated levels of MMP-8 and IL-1β in saliva are linked to periodontal disease progression.	[5,13]
Dental Caries and Immune Response	Higher salivary levels of CD14 and s-IgA are associated with lower caries risk in children.	[17,18]
Salivary Diagnostics in Diabetes	Salivary glucose correlates significantly with blood glucose levels, indicating potential for non-invasive diabetes monitoring.	[19,25,27]
Saliva Testing for Infectious Diseases	Saliva has proven effective for SARS-CoV-2 detection and may be useful for tuberculosis and HIV diagnostics.	[28,29,30]
Salivary Biomarkers for Cardiovascular Diseases (CVD)	Elevated salivary C-reactive protein (CRP) levels correlate with systemic inflammation in CVD patients.	[20,21,23]
Cancer Biomarkers in Saliva	mRNA biomarkers (KRAS, MBD3L2, ACRV1, DPM1) can differentiate pancreatic cancer patients from healthy individuals.	[32,33]
Single-Cell Sequencing in Cancer Research	Single-cell sequencing has identified variant surface glycoproteins (mVSGs) as targets for vaccine development against Trypanosoma brucei.	[61,64]
Proteomics and Metabolomics for Disease Detection	Proteomic analysis helps distinguish between normal and pathological states in systemic diseases.	[53,54,58,59]
Raman Spectroscopy (RS) for Cancer Diagnosis	RS and surface-enhanced RS (SERS) detect molecular alterations in saliva linked to nasopharyngeal carcinoma and OSCC.	[69,73,75]
SELDI-TOF-MS for Biomarker Detection	This technique detects low-molecular-weight proteins in saliva, improving early cancer detection.	[78,80]
Heavy Metals in Saliva	Elevated nickel and chromium levels in saliva due to dental appliances may lead to oxidative stress and inflammatory responses.	[43,46,48]
Saliva Kits for Disease Screening	Saliva kits for HIV/AIDS detection have accuracy rates exceeding 99% in some studies.	[65,66]

Limitations

While salivary diagnostics offer numerous benefits, this review acknowledges certain limitations. The variability in salivary composition due to factors such as age, gender, diet, and medication can impact the reliability and accuracy of results. Additionally, the lack of standardized protocols for saliva collection, handling, and analysis may affect the consistency of findings across different studies. Furthermore, the relatively lower concentration of certain biomarkers in saliva compared to other bodily fluids, such as blood, may require more sensitive detection technologies, which are not yet widely accessible in clinical settings.

Prospects

Despite these limitations, the future of salivary diagnostics remains promising. Continued advancements in analytical technologies, such as next-generation sequencing, proteomics, and metabolomics, are expected to enhance the sensitivity and specificity of saliva-based tests. There is also significant potential for saliva diagnostics to be integrated into personalized medicine, offering non-invasive and cost-effective tools for early disease detection and monitoring. Research into more robust, standardized protocols for saliva collection and analysis will be critical in establishing saliva as a mainstream diagnostic tool in both dental and systemic disease management.

## Conclusions

This comprehensive literature review highlights the growing importance of salivary tests in modern dental practice. Salivary biomarkers have shown promise in the diagnosis, prognosis, and management of various oral and systemic health conditions. However, to fully realize the potential of salivary tests, further research is needed to address the challenges related to variability, standardization, clinical validation, and integration with existing diagnostic tools. The non-invasive nature of saliva collection, combined with its cost-effectiveness and ease of use, positions saliva as a viable alternative to traditional diagnostic methods, particularly for patients who may be apprehensive about invasive procedures. As the field of salivary diagnostics continues to evolve, dentists and healthcare professionals should stay informed about the latest advancements and consider incorporating salivary tests into their clinical decision-making processes, where appropriate. The use of saliva as a diagnostic tool is beneficial not only in the detection of dental caries and its progression but also in the monitoring of periodontal diseases, oral cancers, and systemic illnesses such as diabetes and CVDs.

The collection of saliva is a simple procedure that does not require complex equipment, making it suitable for point-of-care applications and at-home monitoring. Moreover, advances in technologies such as proteomics, metabolomics, and genomic analyses have expanded the potential of saliva-based testing, enabling more precise identification of biomarkers associated with various health conditions. However, the successful implementation of these advancements in clinical practice requires collaboration across multiple disciplines. Ongoing collaboration between researchers, clinicians, and industry partners will be crucial in refining diagnostic techniques, developing robust protocols, and ensuring the accessibility of salivary diagnostics on a global scale. Ultimately, integrating salivary diagnostics into routine dental and medical care can lead to earlier disease detection, personalized treatment approaches, and improved patient outcomes. To achieve this, efforts must be made to bridge gaps in research, education, and clinical adoption, thereby solidifying the role of salivary diagnostics as a cornerstone of preventative and precision healthcare.
